# Meralgia Paresthetica and Femoral Acetabular Impingement: A Possible Association

**DOI:** 10.4021/jocmr468w

**Published:** 2010-12-11

**Authors:** Aiesha Ahmed

**Affiliations:** Neurology Department, Penn State College of Medicine, Hershey, PA 17033, USA. Email: aiahmed1@yahoo.com

## Abstract

Meralgia paresthetica consists of pain and dysesthesia in the anterolateral thigh. Etiology is divided into spontaneous and iatrogenic causes. To my knowledge this has never been attributed to femoral acetabular impingement. This case highlights the presence of lateral femoral cutaneous neuropathy in the setting of femoral acetabular impingement syndrome thus raising the possibility of an association.

**Keywords:**

Femoral acetabular impingement; Lateral femoral cutaneous nerve; Dysesthesia; Nerve conduction studies

## Introduction

Meralgia paresthetica or lateral femoral cutaneous neuropathy is a neurological disorder due to the entrapment of the lateral femoral cutaneous nerve with subsequent numbness and/or burning pain on the anterolateral aspect of the thigh. Meralgia paresthetica is categorized as spontaneous or iatrogenic. Spontaneous causes include different mechanical factors. This case report suggests femoral acetabular impingement syndrome to be considered as one of the possible spontaneous causes of meralgia paresthetica.

## Case Report

A 33-year-old man came for evaluation of pain in his right thigh. His symptoms started approximately three years ago. He suffered from two kinds of pain, one was deep-seated right hip pain, which was insidious in onset and got gradually progressive and was diagnosed as femoral acetabular impingement syndrome. The second kind of pain was described by him as burning pain on the anterior and lateral aspect of his thigh. The skin of his right thigh was hypersensitive to touch. The burning pain started within a month of the deep seated hip pain. He denied any weakness. However, he was limited in his activity due to the pain. He had no back pain. He denied any bowel or bladder incontinence. His past medical and family history were unremarkable. He is a truck driver by profession and denied smoking, drinking and use of illicit drugs. He recently was started on Cymbalta 30 mg daily for the burning pain. On exam his vitals were stable. He was of normal body habitus and reported no recent weight changes. He weighed 200 pounds and his height was 6 feet. Heart sounds were audible and chest was clear to auscultation. His cranial nerve exam was unremarkable. Motor examination showed full strength for neck flexion and extension as well as the upper and lower extremity muscles proximally and distally. No fasciculation or atrophy were noted anywhere. No trophic skin changes were noted. His tone was normal throughout. His reflexes were 2+ in the upper and lower limbs bilaterally. Babinski sign was mute bilaterally. No Hoffman sign was noted. Sensory examination showed hyperesthesia in L2, L3 and L4 distribution of the right thigh, otherwise normal pinprick elsewhere. Vibration and position were intact symmetrically. He was able to get up from a seated position with his arm folded across his chest. He had no dysmetria. His casual gait was normal. He could stand on his heels and on his tiptoes. He was able to maintain his stance on Romberg test. Work-up that was done includes magnetic resonance imaging (MRI) on right thigh which showed a well defined fluid collection between the femoral condyle and iliotibial band and was suggested to be responsible for mild iliotibial band friction syndrome. Lumbosacral spine MRI was unremarkable. Right hip MRI showed labral cartilage being 'pinched' between the rim of the socket and the anterior femoral head-neck junction with superior and laterally torn labrum. Medial femoral head osteophyte was also noted. Based on the imaging studies, he was diagnosed with femoral acetabular impingement syndrome. Two weeks prior to the presentation at our clinic, he had arthroscopic debridement of the torn labrum, arthroscopic osteochondroplasty of pincer, and cam lesion. Subsequent to surgery he noted improvement in his deep seated hip pain up to 90%. However, the burning pain had remained unchanged. A right lateral femoral cutaneous neuropathy was suspected and an electrodiagnostic study was performed (Table 1). There was electrophysiologic evidence suggestive of a right lateral femoral cutaneous neuropathy as no response was seen on the right side compared to normal response on the left side. Lumbar paraspinal and femoral nerve innervated muscles were normal on the needle exam. Patient was referred to pain clinic for nerve block.

**Table 1:** Nerve Conduction Studies in a Patient With Meralgia Paresthetica

Nerve	Sensory Latency (ms)	Sensory Amplitude (V)	Motor Distal Latency (ms)	Motor Distal Amplitude (mV)	Conduction Velocity (m/s)	F-Wave Conduction Velocity (m/s)
Peroneal			R: 5.1 L: 5.3 (< 6.1)	R: 5.8 L: 6.2 (> 3.0)	R: 51 L: 48 (> 41)	
Tibial			R: 4.8 L: 5.1 (< 6.1)	R: 5.3 L: 5.1 (> 2.0)	R: 55 L: 52 (> 41.0)	
Sural	R: 4.0 L: 3.9 (< 4.2)	R:17 L:12 (> 6)			R: 50 L: 52 (> 40)	
Superficial peroneal	R: 3.2 L: 3.0 (< 4.4)	R: 7 L: 10 (> 6)			R: 54 L: 51 (> 40)	
Lateral femoral cutaneous	R: NR L: 3.3 *	R: NR L: 6			R NR L: 43	

NR = no response. Normal values are given in parentheses. *There is no set normal value range.

## Discussion

Meralgia paresthetica or lateral femoral cutaneous neuropathy is a neurological disorder due to the entrapment of the lateral femoral cutaneous nerve with subsequent signs and symptoms in the distribution of the nerve. The symptoms are described as numbness, burning pain and/or dysthesia on the anterolateral aspect of the thigh. Symptoms may worsen on walking, standing or extension of hips [1]. The lateral femoral cutaneous nerve is an entirely sensory nerve that is usually derived from lumbar nerve roots L1, L2 and L3 in different combinations [2]. Meralgia paresthetica can be categorized as spontaneous or iatrogenic. Spontaneous causes include mechanical factors such as obesity and increased intra-abdominal pressure, degeneration of pubic symphysis, limb length discrepancy, and pelvic crush fractures. Metabolic causes will also come under the spontaneous category and include lead poisoning, diabetes, alcoholism and infections such as leprosy. Iatrogenic causes include orthopedic procedures such as pelvic osteotomy, spine surgery, iliac crest bone grafting, total hip replacement and different types of laparoscopic procedures [3]. Most commonly, the lateral femoral cutaneous nerve travels under the psoas major muscle and enters the thigh by crossing the groin under the inguinal ligament, after which it pierces the fascia lata and then divides into its anterior and posterior branches. There are anatomical variations in the course of the lateral femoral cutaneous nerve which may be responsible for the susceptibility of the nerve to injury due to the above mentioned etiologies [3, 4]. There are hypotheses in the literature that mentions compression of the LFCN triggered by repetitive or continuous contraction and stretching of the iliopsoas and sartorius muscles in conjunction with anatomic abnormalities [4]. Similarly, pelvic tilt and limb discrepancy are hypothesized to tense the inguinal ligament resulting in compression of lateral femoral cutaneous nerve [5].

To the best of my knowledge this is the first reported case of lateral femoral cutaneous neuropathy caused by femoral acetabular impingement. Femoral acetabular impingement is of two types. Pincer-type involves abnormal morphology of acetabulum and cam impingement is the result of the contact between an abnormally shaped femoral head which can cause avulsion of labrum. The femoral acetabular impingement can cause repetitive impact on the femoral neck against the acetabular labrum [6]. Patient activity level seems to be an important factor in the pathogenesis of this disorder, and athletes who participate in sports that require repetitive hip flexion and abduction, are at increased risk for symptomatic FAI [7]. It is plausible to speculate that anatomical variability of the lateral femoral cutaneous nerve, contraction of the inguinal ligament, stretching of the iliopsoas muscle, and proximity to morphologically abnormal hip joint as well as pelvic tilt may all be contributory factors to the development of meralgia paresthetica in this patient. There are numerous causes of spontaneous meralgia paresthetica listed in the literature. In the appropriate clinical setting, femoral acetabular impingement should be considered when evaluating patients with spontaneous meralgia paresthetica.
